# Apothecaries

**Published:** 1850-01

**Authors:** 


*Apothecaries.*—We commend to the notice of our readers, the advertisement of W. King, jr., in the advertising sheet of this number. Medicines sold by this gentleman, and by Mr. A. I. Mathews, of this city, are selected and prepared with great care, having sole regard to purity and accuracy. Moreover, these gentlemen are entitled to the respect and consideration of the profession, for having discarded from their shops all patent nostrums, confining their attention exclusively to the legitimate business of the Druggist and Apothecary.

